# Optimizing Cardiac Rehabilitation in Heart Failure: Comprehensive Insights, Barriers, and Future Strategies

**DOI:** 10.3390/medicina60101583

**Published:** 2024-09-26

**Authors:** Francisco Epelde

**Affiliations:** 1 Internal Medicine Consultant, Hospital Universitari Parc Taulí, Sabadell, 08208 Sabadell, Spain; fepelde@gmail.com; 2 Medicine Department, Universitat Autonoma of Barcelona (UAB), 08193 Barcelona, Spain

**Keywords:** heart failure, cardiac rehabilitation

## Abstract

Heart failure (HF) is a prevalent cardiovascular disease associated with significant morbidity, mortality, and healthcare costs. Cardiac rehabilitation (CR) is a structured, multidisciplinary intervention that has been proven to improve functional capacity, reduce hospital readmissions, and enhance the quality of life in HF patients. Despite strong clinical evidence and guideline endorsements, CR remains underutilized in this population. This paper provides a comprehensive review of the role of CR in HF, focusing on exercise-based rehabilitation, psychosocial support, and education. It also explores the barriers to CR implementation, such as patient-related factors, provider-related issues, and systemic challenges. Additionally, we propose future strategies to increase CR uptake, including personalized CR programs, telehealth innovations, and integrating CR into routine HF care pathways. By addressing these challenges and implementing these strategies, healthcare systems can optimize CR delivery and improve outcomes for HF patients.

## 1. Introduction

Heart failure (HF) is a global health burden affecting over 26 million people worldwide [1]. Characterized by the heart’s inability to pump blood effectively, HF leads to reduced functional capacity, recurrent hospitalizations, and a decline in quality of life. Despite advances in pharmacological and device-based therapies, HF continues to be associated with high morbidity and mortality [2]. Consequently, non-pharmacological interventions, such as cardiac rehabilitation (CR), have gained importance in managing HF.

CR is a comprehensive intervention that combines exercise training, education, and psychosocial support to enhance cardiovascular health and improve functional outcomes [3]. While traditionally associated with post-myocardial infarction (MI) care, the role of CR in HF has gained increasing recognition over the past decade. Clinical trials and meta-analyses have consistently shown that CR improves functional capacity, reduces symptoms, and enhances the quality of life in HF patients [4]. Key trials, including HF-ACTION and ExTraMATCH II, have demonstrated significant reductions in mortality and hospitalizations associated with exercise-based CR [5,6].

The American Heart Association (AHA) and American College of Cardiology (ACC) guidelines recommend CR for patients with chronic stable HF, particularly those with reduced ejection fraction (HFrEF) [7]. Similarly, the European Society of Cardiology (ESC) endorses CR as a critical component of HF management [8]. Despite these recommendations, CR participation rates among HF patients remain low, with less than 30% of eligible patients enrolling in CR programs [9].

This paper aims to provide a comprehensive review of the evidence supporting CR in HF, highlight the barriers to its implementation, and propose future strategies to optimize CR delivery. By addressing the challenges that hinder CR participation, healthcare systems can improve outcomes for HF patients and reduce the burden of this debilitating condition.

## 2. The Role of Cardiac Rehabilitation in Heart Failure

CR is a multidisciplinary intervention designed to enhance cardiovascular health through exercise training, education, and behavioral changes [10]. In the context of HF, where patients often experience exercise intolerance and skeletal muscle dysfunction, CR offers numerous benefits, including improved aerobic capacity, reduced symptoms, and enhanced quality of life [11]. This section will explore the key components of CR in HF, focusing on exercise training, psychosocial support, and education.

### 2.1. Exercise Training in CR for HF

Exercise training is the cornerstone of CR and has been shown to provide significant benefits for HF patients. HF is often associated with exercise intolerance due to impaired cardiac output, skeletal muscle dysfunction, and abnormalities in peripheral circulation [12]. As a result, patients with HF often experience reduced exercise capacity, which contributes to their overall poor quality of life. Exercise-based CR aims to counteract these effects by improving cardiovascular fitness, enhancing skeletal muscle function, and promoting overall physical activity [13].

Numerous studies have demonstrated the positive impact of exercise training on HF outcomes. A meta-analysis by Taylor et al. found that exercise-based CR significantly reduced all-cause mortality and hospital admissions in HF patients [14]. The Exercise Training in Chronic Heart Failure (ExTraMATCH II) study also showed that aerobic exercise reduces mortality and HF-related hospitalizations [15]. Additionally, resistance training, when combined with aerobic exercise, improves muscle strength, endurance, and overall physical function in HF patients [16].

The mechanisms underlying the benefits of exercise training in HF are multifactorial. Exercise has been shown to improve endothelial function, enhance autonomic regulation, and increase skeletal muscle oxidative capacity [17]. These physiological adaptations contribute to improved exercise tolerance, reduced symptoms, and enhanced quality of life in HF patients.

One of the landmark trials supporting the use of exercise-based CR in HF is the HF-ACTION trial. This large, multicenter randomized controlled trial involved over 2300 patients with HFrEF and evaluated the impact of exercise training on mortality and hospitalization [18]. The results showed that patients who participated in exercise-based CR experienced significant improvements in exercise capacity, quality of life, and reductions in both mortality and hospitalizations [19]. The HF-ACTION trial remains one of the most influential studies in demonstrating the effectiveness of CR in HF.

Despite the overwhelming evidence supporting the benefits of exercise training in HF, participation rates in CR programs remain low. Several factors contribute to this underutilization, including patient-related barriers, provider-related challenges, and systemic issues within the healthcare system. These barriers will be discussed in detail later in this paper.

### 2.2. Psychosocial Support and Education in CR

In addition to exercise training, CR programs incorporate psychosocial support and education to address the holistic needs of HF patients. Depression, anxiety, and social isolation are common in HF and are associated with worse clinical outcomes [20]. Psychosocial interventions within CR, such as stress management, counseling, and social support, can help reduce these psychological burdens and improve overall well-being [21].

Depression, in particular, is a significant predictor of poor outcomes in HF patients, including increased mortality and hospitalizations [22]. Psychosocial interventions in CR aim to alleviate depressive symptoms and promote positive coping strategies, which can enhance adherence to medical therapy and improve quality of life [23]. Studies have shown that patients who receive psychosocial support within CR programs are more likely to experience improvements in mood, reduce their risk of hospitalization, and achieve better long-term outcomes [24].

Education is another critical component of CR, focusing on self-care, medication adherence, and lifestyle modifications [25]. Patients who are well-informed about their condition and understand the importance of adhering to treatment plans are more likely to engage in behaviors that promote heart health, such as regular exercise, a heart-healthy diet, and medication compliance [26]. Education provided in CR programs empowers patients to take an active role in managing their condition, which can lead to better outcomes and fewer hospital readmissions [27].

### 2.3. Special Considerations for HF Patients in CR

HF is a complex and heterogeneous condition, and not all patients may benefit equally from traditional CR programs. Some HF patients, particularly those with advanced disease, may require more tailored interventions that take into account their unique clinical characteristics [28]. For example, patients with severe HF or those awaiting heart transplantation may need more closely supervised exercise regimens and specialized care to ensure safety and efficacy [29]. Personalizing CR programs to meet the specific needs of different HF populations is an important consideration for optimizing outcomes.

CR programs for HF patients should also account for the potential presence of comorbidities, such as chronic obstructive pulmonary disease (COPD), diabetes, or obesity, which can further complicate exercise tolerance and rehabilitation [30]. Multimorbidity is common in HF patients, and addressing these comorbid conditions within the context of CR can enhance the overall effectiveness of the intervention [31].

## 3. Barriers to Cardiac Rehabilitation in Heart Failure

Despite the clear benefits of CR for HF patients, participation rates remain suboptimal. Several barriers contribute to the underutilization of CR in this population, including patient-related factors, provider-related challenges, and systemic issues within the healthcare system. Understanding and addressing these barriers is crucial to improving CR uptake and ensuring that more HF patients benefit from this life-saving intervention.

### 3.1. Patient-Related Barriers

Patient-related factors are one of the most significant barriers to CR participation. Many HF patients are unaware of the benefits of CR or may have misconceptions about its purpose and effectiveness [32]. Some patients may perceive CR as unnecessary or believe that they are too frail or ill to engage in exercise-based rehabilitation [33]. These misconceptions can lead to low motivation and reluctance to participate in CR programs.

In addition to misconceptions, logistical challenges such as transportation, work obligations, and caregiving responsibilities can prevent patients from attending CR sessions [34]. HF patients, particularly older adults, may face difficulties traveling to CR facilities, especially if they live in rural areas or have limited access to transportation [35]. Financial constraints, such as the cost of transportation, copayments, or lack of insurance coverage, can also pose significant barriers to CR participation [36].

Another important consideration is the physical and psychological burden of HF itself. HF patients often experience fatigue, shortness of breath, and other symptoms that can make it difficult to engage in exercise or attend regular CR sessions [37]. The fear of exacerbating symptoms or experiencing adverse events during exercise may deter some patients from participating in CR programs [38]. Additionally, depression and anxiety, which are common in HF, can reduce motivation and adherence to CR [39].

To address these patient-related barriers, it is essential to provide education about the benefits of CR and to tailor CR programs to meet the specific needs and preferences of each patient [40]. Patient-centered approaches, such as home-based or telehealth CR programs, can help overcome logistical challenges and improve access to rehabilitation services [41]. Additionally, providing psychosocial support and addressing mental health concerns within CR programs can enhance motivation and adherence [42].

### 3.2. Provider-Related Barriers

Healthcare providers play a crucial role in the referral and delivery of CR services. One significant barrier is the lack of awareness and knowledge among healthcare providers about the benefits of CR for HF patients [43]. Some providers may not be familiar with the latest evidence or guidelines recommending CR for HF, leading to missed opportunities for referral [44].

Continuing medical education (CME) programs and targeted initiatives to increase provider awareness about CR can help address this barrier [45]. Additionally, incorporating CR education into medical training and providing resources to support healthcare providers in making CR referrals can improve utilization rates [46].

Another challenge is the variability in CR program availability and quality. In some regions, CR programs may be limited or lack the necessary resources to provide comprehensive care [47]. Ensuring that CR programs are adequately resourced and accessible to all patients is essential for improving participation rates [48].

### 3.3. Systemic Barriers

Systemic factors also play a role in the underutilization of CR. Fragmented healthcare systems and lack of integration between different levels of care can hinder the delivery of CR services [49]. For example, patients may experience delays in receiving CR referrals or face difficulties navigating the healthcare system to access appropriate services [50].

To address these systemic barriers, it is important to integrate CR into routine HF care pathways and to improve coordination between primary care providers, cardiologists, and CR programs [51]. Streamlining referral processes and ensuring that CR services are included in standardized treatment protocols can enhance access to rehabilitation services [52].

## 4. Future Directions for Cardiac Rehabilitation in Heart Failure

To enhance the delivery and effectiveness of CR for HF patients, several future directions should be considered. These include the development of personalized CR programs, the expansion of telehealth and home-based CR, and the integration of new technologies and medications into CR strategies.

### 4.1. Personalized Cardiac Rehabilitation Programs

Personalizing CR programs to meet the specific needs of different HF populations is a key strategy for improving outcomes. This includes tailoring exercise regimens to accommodate patients with varying degrees of functional impairment and comorbidities [53]. Advances in technology, such as wearable fitness devices and AI-driven exercise regimens, offer opportunities to create more individualized CR programs that can adapt to patients’ needs and progress [54].

Incorporating new medications, such as SGLT2 inhibitors, into CR strategies may also enhance the effectiveness of rehabilitation [55]. These medications have shown promise in improving HF outcomes and may complement the benefits of CR by addressing underlying pathophysiological mechanisms [56]. Exploring the potential interactions between new drugs and CR interventions can lead to more effective and personalized treatment approaches. A recent study highlights the effects of SGLT2 inhibitors on atrial fibrillation, suggesting their potential pleiotropic benefits in CR [57].

### 4.2. Telehealth and Home-Based Cardiac Rehabilitation

Telehealth and home-based CR programs have gained prominence as alternative methods for delivering rehabilitation services. These approaches offer several advantages, including increased accessibility, convenience, and the ability to reach patients who may otherwise be unable to attend center-based programs [58]. However, the effectiveness of telehealth CR compared to traditional programs and its cost-effectiveness need further investigation [59].

Expanding the discussion to include challenges associated with telehealth implementation, such as access to technology, digital literacy, and the need for telemonitoring infrastructure, can provide a more comprehensive view of this approach [60]. Addressing these challenges and developing strategies to overcome them will be crucial for the successful implementation of telehealth CR programs.

### 4.3. Integration of New Technologies and Medications

The integration of new technologies and medications into CR programs has the potential to enhance personalization and effectiveness. Wearable devices and AI-driven tools can provide real-time feedback and adjust exercise regimens based on individual patient data [61,62,63]. Additionally, new medications, such as SGLT2 inhibitors, may offer additional benefits when incorporated into CR strategies [64,65,66,67,68,69,70]. Exploring these innovations and their impact on CR can contribute to more effective and tailored rehabilitation programs.

## 5. Conclusions

Cardiac rehabilitation plays a critical role in the management of HF, providing significant benefits through exercise training, psychosocial support, and education. Despite the evidence supporting its efficacy, CR remains underutilized due to various barriers. Addressing these barriers and implementing strategies to improve CR delivery, such as personalized programs, telehealth innovations, and the integration of new technologies and medications, is essential for optimizing outcomes for HF patients. By advancing these initiatives, healthcare systems can enhance the effectiveness of CR and improve the quality of life of individuals living with HF.
